# Analysis of losses in physiotherapy students during the COVID-19 pandemic: a phenomenological approach

**DOI:** 10.1186/s40359-024-01848-w

**Published:** 2024-06-14

**Authors:** Marta Terrón-Pérez, Sara Cortes-Amador, Juan Bautista Portolés-Simeó

**Affiliations:** 1 Department of Nursing, University of Valencia, Valencia, Spain; 2 Department of Physiotherapy, University of Valencia, Valencia, Spain

**Keywords:** Grief, COVID-19, Students, Physical therapy specialty, Education, Universities

## Abstract

**Background:**

During the COVID-19 pandemic, young people have experienced numerous personal losses across various aspects, impacting their quality of life. This study aimed to explore and analyze the losses experienced by physiotherapy students during the first year of the COVID-19 pandemic.

**Methods:**

A qualitative phenomenological study was conducted using an open-format exercise carried out during the Clinical Specialties class from February to May 2021. Thirty-four (83% female) third-year physical therapy students participated. ATLAS.ti software was used for the analysis and coding by three researchers.

**Results:**

Analysis of the categories revealed various losses experienced by the participants, including losses in psychological well-being, physical health, the social sphere (friendships, relationships with partners and family members, and experiences of death), spiritual losses (loss of freedom and identity), leisure time (travel, recreational activities and physical exercise), and different losses related to university studies (motivation and enthusiasm and clinical practices).

**Conclusion:**

The COVID-19 pandemic has led to significant losses among physiotherapy students, with losses in the social sphere being the most prevalent. This study can serve as a foundation for developing resources aimed at enhancing the well-being of physiotherapy students, promoting optimal academic performance, improving self-care, and reducing psychosocial problems.

## Background

The COVID-19 pandemic has affected more than 550 million people worldwide, resulting in more than 6.3 million deaths [1, 2]. This pandemic has significantly impacted various aspects of life across all countries, including social, economic, and quality of life dimensions [3]. Young adults, in particular, have been extensively studied during the pandemic. They have experienced psychological challenges such as stress, anxiety, depression, and suicidal thoughts [4]. Loneliness is prevalent among these individuals [5]. Additionally, there has been an increase in obesity, overweight [3], and sedentary lifestyles [6].

These circumstances have led to numerous personal losses.

Loss can be defined as any damage to the emotional, material, or symbolic resources with which the person has an emotional bond [7]. Loss can occur due to disability, changes in work or home, the death of loved ones, etc. [8]. The process that triggers the loss is grief [9]; this process does not indicate any pathology, but affective, cognitive, behavioral and physiological alterations can arise, such as sadness, disbelief, crying or insomnia [10]. It is crucial for health professionals to understand these processes [11].

Various studies emphasize the importance of qualitative research and call for designs that consider subjective narratives to analyze the impact of COVID-19 [12, 13]. While qualitative studies have been conducted with students from other disciplines [14], there is a lack of research specifically focusing on physiotherapy students and their losses from a global perspective. Therefore, it was decided to conduct the following study with the objective of analyzing and explaining the losses experienced by physiotherapy students during the first year of the COVID-19 pandemic.

## Methods

### Study design and recruitment

A qualitative phenomenological study was conducted. The study focused on third-year physiotherapy students at the University of Valencia enrolled in the Clinical Specialties II course. The data were collected from February to May 2021. Participants were selected through convenience sampling after the academic year ended. All students who wished to participate were included, with no exclusion criteria.

### Procedure

Classroom work was proposed, consisting of answering a question with an open-ended free response. The exercise proposed was the following: “Develop a list of duels or personal losses this year that have impacted you the most”. At the time of this exercise, the students were unaware that their responses would be used for research purposes.

### Analysis

The data analysis involved a process of transcription and pairwise coding. Each participant was anonymized and assigned a code number (Subjects 1 through 34; in the work, abbreviated as S1, S2, S3, etc.). The coding and categorization tasks were carried out by two researchers, with a third reviewer facilitating the triangulation of the information. The procedure described by Colaizzi [15] was followed for the treatment of information. First, in-depth reading of the texts of the participants (A), followed by extraction of identification texts (B), these texts were assigned representative codes (C), and the texts were grouped by meaning into categories (D). Finally, the phenomenon of the study was described by interpretive writing (E). For analysis of the texts, ATLAS.ti 22 software was used. Data saturation was reached when both principal investigators observed redundancy in participants' responses during the analysis phase, and no new categories emerged [16].

### Ethics

The research project was presented to and approved by the Ethics Committee of the University of Valencia (Protocol Number: 1782311) in accordance with the principles outlined in the Declaration of Helsinki. Before integrating the students' work into the study, explicit authorization was obtained from all participants through informed consent procedures. Importantly, every student included in the study agreed to participate.

## Results

**Fig. 1:**
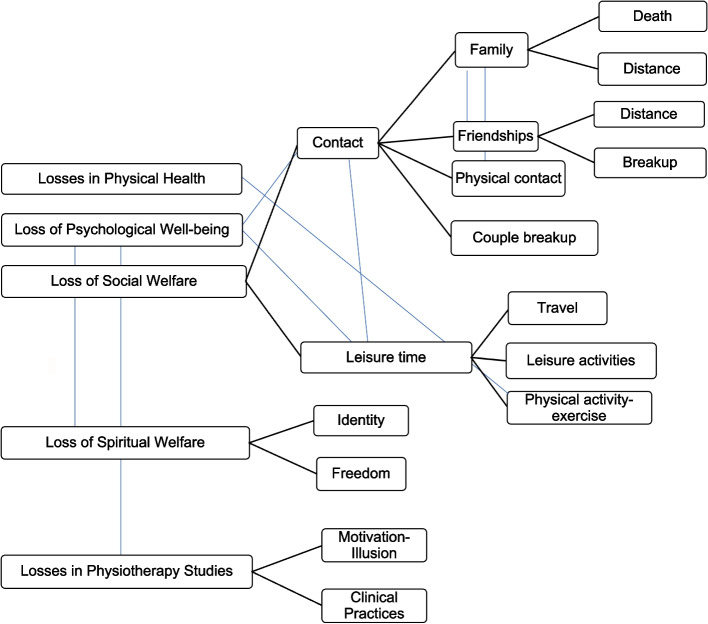
Category tree

A total of 34 students (83% women) out of the 160 who completed the assignment participated, with an average age of 23.3 years. The participation of 34 subjects was sufficient to reach data saturation. After the analysis of the categories, different losses experienced by the participants were identified. Figure 1 shows the themes and subthemes identified.

### Theme 1. Losses in physical health

Three different situations were identified:

Subjects who suffered from the disease mildly:

“Loss of taste and smell, I was infected with COVID-19, also suffering from constant headaches. The loss of pleasure from eating was a significant loss for me. To date, I have recovered part of these senses, but not 100%” S7.

On the other hand, those who have not been able to receive therapy because they cannot travel to their place of treatment:

“This year I have not been able to go to Germany as usual for the past 10 years to be able to treat my disease. Germany is… where I can improve my disease” S13.

Or for delaying a pending surgery:

“My knee injury (torn meniscus). This event this summer has been for me a real odyssey of feelings and sensations after my surgery was delayed” S11.

The third was the losses due to persistent COVID-19:

“Loss of general well-being: apparently, I am one of the people who has the damn persistent COVID… I continue to suffer continuous headaches and episodes of tachycardia, which also cause me anxiety and low mood” S7.

### Theme 2. Loss of psychological well-being

At the psychological level, the loss of mental health caused alterations in students’ anxiety and depression:

“As time went by, it continued to have an impact on my mind until in the summer I exploded and I was plunged into terrible anxiety and a little depression…” S21.

In addition, negative psychological responses were found, such as insomnia, worry or feeling overwhelmed:

“I have spent days crying, having to study, with insomnia, without being able to leave the house to relax, without hope to live, I felt that the house was caving on me” S19.

Additionally, losses related to mental stability or loss of enthusiasm for life:

“As for my mental health, it has been by far the worst year I have experienced, the most unstable I have felt and the least I have understood myself. I had no illusion about anything, and every day seemed the same” S26.

### Theme 3. Loss of social welfare

#### Subtheme. Loss of contact

- Loss of family contact due to death

In the present study, 14 of the 34 participating students died from a loved one:

“The death of my grandmother. For me, she was one of the most important people in my life with whom I have shared practically my entire life…” S23.

Many of the participants did not have the opportunity to say goodbye to their loved ones:

“The loss of my uncle who died from causes unrelated to the coronavirus in May 2020 and a mass and “decent” farewell could not be held at the time” S16.

Other notable losses that have been noted are those of household animals:

“The loss of my cat in the middle of confinement, since he was at home and I was in the student apartment, I could not say goodbye to him” S26.

b) Loss of family contact due to distance

Not being able to be with the family has been one of the greatest heartfelt losses, both in the day-to-day:

“The fact of not being able to go to a family meal with my uncles, grandparents, cousins… It has been one of the most important losses for me” S32.

As in important celebrations:

“In addition, the loss of family gatherings at Christmas is a very important loss because you do not know if we will all be at the next holidays” S14.

Another aspect detected was the loss of time with grandparents:

“One of the most important losses has been the time lost with people close to me, especially with my grandmothers who are older people and I feel that the time I have with them is getting shorter” S22.

c) Loss of contact with friends due to distance

Losses were found regarding social and friendship relationships during the pandemic:

“The loss of social relationships, I have not seen my lifelong friends for almost 3 months and I miss our relationship because, no matter what they say, a video call is not the same as going out to eat anywhere” S32.

A direct relationship was obtained between the loss of social relationships and the mental health of the students:

“I am a very sociable person, and the fact of not being able to meet my friends or not wanting to do it for prevention has caused me moments of loneliness, sadness, anxiety and despair” S11.

d) Loss of friendship due to breakup

Several situations were found in which a breakup in the friendship relationship occurred:

“Losing a relationship with a childhood friend” S18; “I could begin with the loss of a relationship with those I considered lifelong friends” S29.

e) Loss of partner contact due to breakup

Another aspect to mention was couple breakups:

“The most important loss and the one that has changed my life the most, the breakup with my partner. I put it in this position because it is the one that hurts me the most since it is the only loss that I will probably never recover” S17.

f) Loss of physical contact

The pandemic has allowed investigating an exceptional situation that in normal conditions would not have occurred, the absence of physical contact:

“Not being able to have the same physical contact between us to the point of not greeting each other with two kisses or some gesture of affection” S5.

The students experienced a lack of contact in a negative way:

“Begging for the warmth of the family, more than ever. However, they took it from us again; they took it away again” S9.

#### Subtheme Loss in Leisure Time

- Loss of travel

In the group studied, the loss of trips was also present:

“I had to cancel a trip to Kenya that I had scheduled for half a year” S22.

With respect to shorter trips, a relationship was found with an emotional and affective component:

“The perimeter closure, which prevents you from not only traveling but visiting your loved ones” S5.

- Loss of leisure activities

For the young population, the effect has been very negative, and many comments have been generated about losses in leisure activities:

“However, the greatest loss I have had in general has been social life, going out with my friends, spending time away from home, making plans, etc.…” S31.

Generating pain and suffering in affected people:

“While I want to recover that social life, that joy, that desire to communicate with people, I cannot do it, it is like a paradox, and I consider it a problem” S19.

In many cases, the losses were related to simple activities:

“Just going for a drink with my friends or going for a walk at 12” S20.

b) Loss of physical activity and physical exercise

It is worth noting the losses of physical activity (PA) or physical exercise (PE):

“First, the loss of sports, of basketball and that due to the pandemic has not been resumed today. It was a liberation for me, in addition to being a fun way to keep fit and enjoy with my classmates” S17.

The loss of PA and PE has had physical, social (loss of team sports) and psychological consequences:

“I got frustrated because for me doing sports was an escape, it helped me relax, it reduced my stress, it helped me to forget my routine and even my problems…” S32.

### Theme 4. Loss of Spiritual Welfare

#### Subtheme. Loss of identity

Another source of discomfort was the anguish in the loss of identity:

“I escape from my reality to be able to find the origin of pain, the loss of self, the final climax to a series of minor losses that made my mental structure break like a fragile glass” S3.

Loneliness and isolation were found to be measures of protection against this problem:

“I am not the person I used to be; I prefer to be in my quiet room watching a series without talking to anyone” S19.

#### Subtheme. Loss of freedom

In this category, the concept of freedom is understood in a practical way, with the choice of being able to occupy spaces freely:

“… Loss of freedom, loss of the possibility of going where you want, when you want, with the people you want… One of the most important and one of those that have marked me the most” S10.

Generating a loss of control in people's lives:

“I had a feeling that I did not manage my life, but that it depended on the measures that were available at all times…” S26.

### Theme 5. Losses in physiotherapy studies

#### Subtheme. Motivation-illusion

A response of demotivation and disappointment in relation to one’s career was found:

“COVID-19 has changed our way of understanding university life; it has stolen part of our academic training and has sown apathy and indifference in the classrooms” S1.

#### Subtheme loss of clinical practice

The experience of the lack of clinical practices also generated negative responses:

“…With the new situation, it will not be possible for me to experience hospital practices this year, and clinical practices will not be as I had imagined” S7.

## Discussion

The results indicate an overall loss of well-being in all aspects. The identified categories align with the health aspects defined by the WHO [17]. Additionally, these losses have led to numerous processes of mourning, with associated alterations such as sadness, loneliness, depression, and anxiety [10].

Regarding physical losses, at the beginning of the pandemic, the symptoms of COVID-19 were not well known. Currently, there are studies focused on this topic [18]. However, few studies have assessed the impact of COVID-19 on surgical waiting lists in Spain. A study by de Pablos & García-Centeno [19] indicated a delay of 7.6 to 19.4% in the waiting time for surgery.

Mental health is one of the most affected aspects in young adults compared to other categories. Stress, depression and anxiety were the most common issues. One review revealed that anxiety affects 32% of the population and that 28% of affected individuals suffer from depressive disorders [20]. Additionally, studies have reported compulsive behaviors, avoidance, deterioration of social function, and dissatisfaction with life [21]. Data referring to negative psychological alterations seem to normalize over time [22]. More studies are needed to assess and monitor mental health problems adapted to the context to facilitate appropriate health measures.

Social losses were the most frequent, generating many categories and subcategories and showing a close relationship with mental health losses.

The high percentages of deaths among the participants are consistent with what occurred in Spain during the pandemic [2]. Not being able to perform a community ritual, not being physically present, experiencing loneliness due to lack of support, or not being able to say goodbye to loved ones [23] have changed the way people cope with losses. These situations may have increased the probability of experiencing complicated grief. It would be necessary to conduct research with standardized instruments to observe whether there has truly been an increase in complicated grief during the COVID-19 pandemic.

In relation to the losses due to the deaths of pets, there is currently a period of humanization of companion animals [24]. Symptoms of grief with pets increase in proportion when they coincide with stressful life events [25], which could explain why during the COVID-19 pandemic, pets have been identified as more anxiety-inducing than in normal situations.

Despite multiple sociological changes, the family continues to be an extremely important factor in Spain [26]. Some losses are related to marked festivities, while others are tied to regular contacts. The loss of these ties implies a decrease in social support and an impact on emotional well-being [20], in addition to an increase in stressors [27]. On the other hand, the lack of contact with grandparents was noted, and it would be interesting to investigate the consequences for a young adult of losing relationships or references, such as their grandparents.

At a university age characterized by the achievement of individual autonomy, constant discovery, growth, and the expansion of relationships, the pandemic has negatively impacted development. Research on university students indicates that a greater number of friendships, quality contact, and satisfactory and frequent electronic communication can be strategies that protect against loneliness [28], in addition to being protective against psychological and mental health problems [5].

The phenomenon of couples during the pandemic has been investigated in cohabitant couples and marriages [29] but not in university students. Individuals who are threatened by constant stress outside of a relationship are more likely to interact negatively with their partners [30], and the COVID-19 pandemic has been a generator of conflicts and stressors [31]. Facing conflict requires personal maturity and quality in the relationship, which can be difficult for young university students to acquire.

In relation to the loss of physical contact, studies have indicated the desire and need for contact for many people and the possibility of regulating stress and anxiety through intimate contact [32]. Slow and affectionate touch provides benefits such as the mitigation of negative emotions or the fostering of bonding between individuals [33]. It would be interesting to implement programs that enhance physical contact between people and generate some of the aforementioned benefits.

During the pandemic, and due to restrictions, there has been an enormous amount of free time, with the particularity that the ability to choose activities by the person was lost, as they could not be done outside of a schedule, from home, or in certain inaccessible establishments [34].

There is a social and psychological need during this time to travel, as well as to enjoy the emotional well-being of knowing other cultures, customs, gastronomy, etc. [35]. There is a direct and indirect relationship between travel and life satisfaction, as well as between emotional well-being and the feeling of happiness or pleasure [36]. Additionally, in challenging situations, leisure activities reduce many factors of stress and anxiety [37], in addition to improving well-being in the university population [38]. Therefore, it could be recommended that this population resume travel and engage in activities that reduce academic stress.

Physical activity (PA) and physical exercise (PE) were among the most frequently mentioned losses. Notably, Spain implemented one of the strictest lockdowns. One possible explanation for these findings is that physiotherapy students typically engage in more hours of PA and PE during their free time than other students [39] due to the significance of physical activity in physiotherapy curricula [40], as well as its importance for treating patients' conditions [41, 42]. These findings are consistent with studies that assessed the level of physical activity among physiotherapy students during the lockdown, revealing a notable reduction in both physical activity levels and energy expenditure [43, 44]. Rodriguez-Larrad [45] observed an increase in sedentary behavior and a decrease in both moderate and vigorous physical activity among students in Spain during the pandemic. Given the myriad benefits of PA and PE for individuals across all demographics [46] and their direct correlation with health, governments should refrain from restricting access to PA and PE in similar situations.

From a classical concept, identity would be that which is maintained in each individual and does not vary over time, its essence. This situation encounters a changing reality, which is constantly evolving and is preyed upon uncertainty [47], such as the period described in this study in the context of a global pandemic.

In relation to the lack of freedom, similar results have been found in other contexts [48]. They related the lack of freedom with a need to return to normality or a feeling of urgency to manage time daily at their convenience. Additionally, it was found that young people (18 to 29 years) could experience more stress and anxiety than other groups due to mobility limitations before other deficiencies [49].

Academic burnout consists of a series of negative psychological manifestations, such as anxiety, depression, and fatigue, that occur due to excessive pressure or lack of interest. Academic burnout syndrome can lead to poor academic performance and health issues [50], as observed in the present study. Disappointment and loss of motivation have been frequently discussed in different university environments [51].

### Limitations of the study

Within the limitations of the study, it is noted that the data analyzed belong to a specific population group (university students) circumscribed to a certain age in a specific context (Valencia), which may not represent other population groups. The sample was not selected because of the greater variability of the profiles.

## Conclusions

The present study identified the losses of physiotherapy students during the COVID-19 pandemic at the biological, psychological, social, spiritual and academic levels. These losses include disruptions to social habits, limitations on freedom and leisure opportunities, affective and emotional challenges, the absence of physical contact or sports activities, and thoughts about the death of family members or relationship breakups. Additionally, concerns about the professional future influenced by a lack of clinical practice were reported.

It is crucial for universities to develop resources that enhance the well-being of healthcare students. These resources are essential not only for optimal academic performance but also for fostering reflective practices, which can promote self-care and reduce psychosocial problems.

## Data Availability

No datasets were generated or analysed during the current study.

## Abbreviations

S: Subject
WHO: World Health Organization
PA: Physical Activity
PE: Physical Exercise
